# Continuing Effect of Cytokines and Toll-Like Receptor Agonists on Indoleamine-2,3-Dioxygenase-1 in Human Periodontal Ligament Stem/Stromal Cells

**DOI:** 10.3390/cells9122696

**Published:** 2020-12-16

**Authors:** Christian Behm, Alice Blufstein, Johannes Gahn, Barbara Kubin, Andreas Moritz, Xiaohui Rausch-Fan, Oleh Andrukhov

**Affiliations:** 1 Competence Center for Periodontal Research, University Clinic of Dentistry, Medical University of Vienna, Sensengasse 2a, 1090 Vienna, Austria; christian.behm@meduniwien.ac.at (C.B.); alice.blufstein@meduniwien.ac.at (A.B.); johannes.gahn@gmail.com (J.G.); barbara.kubin@kabsi.at (B.K.); xiaohui.rausch-fan@meduniwien.ac.at (X.R.-F.); 2 Division of Orthodontics, University Clinic of Dentistry, Medical University of Vienna, Sensengasse 2a, 1090 Vienna, Austria; 3 Division of Conservative Dentistry and Periodontology, University Clinic of Dentistry, Medical University of Vienna, Sensengasse 2a, 1090 Vienna, Austria; andreas.moritz@meduniwien.ac.at

**Keywords:** human periodontal ligament stromal cell, immunomodulation, indoleamine-2,3-dioxygenase-1, cytokines, toll like receptor agonist

## Abstract

Transplanted mesenchymal stem/stromal cells (MSCs) are a promising and innovative approach in regenerative medicine. Their regenerative potential is partly based upon their immunomodulatory activities. One of the most investigated immunomediators in MSCs, such as in periodontal ligament-derived MSCs (hPDLSCs), is indoleamine-2,3-dioxygenase-1 (IDO-1) which is upregulated by inflammatory stimuli, like cytokines. However, there are no data concerning continuing IDO-1 expression in hPDLSCs after the removal of inflammatory stimuli, such as cytokines and toll-like receptor (TLR) agonist-2 and TLR-3. Hence, primary hPDLSCs were stimulated with interleukin (IL)-1β, tumor necrosis factor (TNF)-α, interferon (IFN)-γ, TLR-2 agonist Pam3CSK4 or TLR-3 agonist Poly I/C. IDO-1 gene and protein expression and its enzymatic activity were measured up to five days after removing any stimuli. IL-1β- and TNF-α-induced IDO-1 expression and enzymatic activity decreased in a time-dependent manner after cessation of stimulation. IFN-γ caused a long-lasting effect on IDO-1 up to five days after removing IFN-γ. Both, TLR-2 and TLR-3 agonists induced a significant increase in IDO-1 gene expression, but only TLR-3 agonist induced significantly higher IDO-1 protein expression and enzymatic activity in conditioned media (CM). IDO-1 activity of Poly I/C- and Pam3CSK4-treated hPDLSCs was higher at one day after removal of stimuli than immediately after stimulation and declined to basal levels after five days. Among all tested stimuli, only IFN-γ was able to induce long-lasting IDO-1 expression and activity in hPDLSCs. The high plasticity of IDO-1 expression and its enzymatic activity in hPDLSCs due to the variable cytokine and virulence factor milieu and the temporal-dependent responsiveness of hPDLSCs may cause a highly dynamic potential of hPDLSCs to modulate immune responses in periodontal tissues.

## 1. Introduction

Mesenchymal stem/stromal cells (MSCs) are non-hematopoietic progenitor cells, having self-renewal properties and a multipotent differentiation potential in vitro [1,2]. First detected in the bone marrow, MSC-like cells have been isolated from tissues throughout the human body [3], including dental tissues [4,5,6,7], such as the periodontal ligament [8] or the periapical cyst [9].

Human periodontal ligament derived mesenchymal stem/stromal cells (hPDLSCs) were first isolated as a heterogeneous cell population by Seo et al., in 2004 [8]. They meet the minimal criteria of MSCs, expressing specific mesenchymal surface markers, and having a tri-lineage differentiation potential in vitro [2,10]. Located in the perivascular region in a quiescent and undifferentiated state [11,12], hPDLSCs have the ability to migrate to injured and inflamed periodontal tissues. At this site, they are involved in orchestrating periodontal tissue homeostasis and local inflammatory responses [13,14,15]. Beside their endogenous functions, ex vivo expanded MSCs are a promising therapeutic tool in regenerative medicine [15] and the field of inflammatory disorders [16]. Thus hundreds of clinical studies already investigated MSCs-based treatment approaches [17].

One of the mechanisms discussed to be involved in executing the MSCs’ therapeutic potential is their immunomodulatory potential [18]. Like MSCs isolated from other sources, hPDLSCs influence the on-site immuno-inflammatory state via their mainly immunosuppressive abilities on various immune cells [13,19]. One of the most important immunomodulatory mechanisms is the secretion of paracrine factors, such as indoleamine-2,3-dioxygenase-1 (IDO-1) [13,20]. This enzyme catalysis the rate-limiting reaction of the tryptophan metabolism, triggering the oxidative degradation of L-tryptophan into *N*-formyl-kynurenine, which is further metabolized to L-kynurenine [21,22]. The resulted L-tryptophan depletion inhibits local inflammatory responses [23,24].

In resting hPDLSCs immunomodulatory activities and also IDO-1 expression is generally low. Several environmental factors, such as inflammatory cytokines, strongly increase the production of IDO-1 and consequently boost the immunomodulatory activities of hPDLSCs [25]. These cytokines, such as interleukin (IL)-1β, tumor necrosis factor (TNF)-α and interferon (IFN)-γ, are produced by various immune cells [19,26]. Thus, regulation of IDO-1 expression via cytokines represents an important reciprocal mechanism involved in regulating inflammatory responses. Studies already exist which try to capitalize this cytokine boosted immunomediator expression by ex vivo stimulation of hPDLSCs with cytokines to enhance their immunomodulatory activities and consequently their regenerative potential [27]. Particularly, a recent study shows that ex vivo treatment of hPDLSCs with IFN-γ enhances the efficacy of MSC-based therapies [27]. This could be a potent approach since clinical trials partly report only moderate success of MSC-based therapeutic strategies [17]. Therefore, it is crucial to know how long hPDLSCs can retain the immunomodulatory phenotype after the treatment with inflammatory cytokines. However, this question has never been investigated before.

Periodontitis is characterized by a shift of the oral microbiome homeostasis [28,29], causing an overshot local immune response, which leads to collateral destruction of the host periodontal tissues [30]. During this chronic periodontal inflammation, hPDLSCs are not only exposed to cytokines but also to several exogenous virulence factors such as bacterial lipopeptides or viral components. These toll-like receptors (TLRs) agonists impact the expression of several inflammatory cytokines in hPDLSCs [31,32,33]. However, TLRs agonists might also induce anti-inflammatory, immunosuppressive properties of hPDLSCs, but this question is poorly investigated (reviewed in [19]). TLR-triggered IDO-1 expression could be considered as a potential candidate to mediate such an immunosuppressive effect. Some studies already report activation of IDO-1 production in hPDLSCs, triggered by various TLR agonists such as synthetic TLR2/1 agonist Pam3CSK4 [31,34], but these data are inconsistent. Additionally, there are no data on the effect of viral products, such as the synthetic TLR-3 agonist polyinosinic:polycytidylic acid (PolyI/C), on the IDO-1 expression and activity in hPDLSCs, so far. Periodontal pathogens and their virulence factors are essential drivers of the inflammatory response during periodontal disease progression. Therefore, it is important to know how long Poly I/C- and Pam3CSK4-activated responses persist in hPDLSCs. However, this question has never been explored so far.

Hence, this in vitro study aimed to test the continuing effects of IL-β, TNF-α and IFN-γ as well as of the two TLR agonists, Poly I/C and Pam3CSK4. Additionally, this study aimed to directly compare the contribution of Poly I/C and Pam3CSK4 on the IDO-1 production in hPDLSCs. In particular, primary hPDLSCs were stimulated with IL-β, TNF-α or IFN-γ or with Poly I/C or Pam3CSK4, followed by analyzing their effect on IDO-1 gene and protein levels and on IDO-1 enzymatic activity, one and five days after removing any stimuli.

## 2. Materials and Methods

The Ethics Committee of the Medical University of Vienna approved the isolation of hPDLSCs and the study protocol (EK Nr. 1694/2015, extended 2019). All experiments were conducted according to the Declaration of Helsinki and the Good Scientific Practice Guidelines of the Medical University of Vienna.

### 2.1. Cell Culture

Periodontal ligament tissue was scraped off from the mid-third of the root’s surface of third molars, which were extracted due to orthodontic reasons. All included individuals were periodontally healthy, were aged between 18 and 30 years and gave their informed written agreement before the surgical procedure. Tissue slices were minced using a scalpel and were cultured until hPDLSCs grew out of them. The cells were cultivated in Dulbecco’s modified Eagle’s medium (DMEM, Sigma-Aldrich, St. Louis, MO, USA), which was supplemented with 50 µg/mL penicillin (P, Gibco, Carlsbad, CA, USA), 100 U/mL streptomycin (S, Gibco, Carlsbad, CA, USA) and 10% fetal bovine serum (FBS, Gibco, Carlsbad, CA, USA). They were cultured at 37° Celsius, 5% CO_2_ and 95% humidity. The mesenchymal stromal cell character of the tissue-outgrown cells was verified by analyzing mesenchymal and hematopoietic surface markers’ expression by immunostaining, according to our previous study [35] and the minimal criteria for MSCs defined by the International Society for Cell and Gene Therapy [2,10]. Nevertheless, the isolated cells were a heterogeneous, fibroblast-like population, containing osteoblasts, odontoblasts, fibroblasts and MSCs. Since all these cell types express similar surface markers as the MSCs progenitor population, to data, it is very hard to discriminate and isolate a pure MSCs population [2,10]. hPDLSCs with passage levels between four and seven were used for experiments.

### 2.2. Stimulation Protocol

In total, 250,000 hPDLSCs were seeded per well in 6-well plates using 3 mL DMEM supplemented with 10% FBS and 1% P/S. After 24 h incubation, hPDLSCs were stimulated with one of the following commercially available cytokines or TLRs: 100 ng/mL of human recombinant IFN-γ [25,36] (PeproTech, Rocky Hill, SC, USA), 10 ng/mL human recombinant TNF-α [25,37] (PeproTech, Rocky Hill, SC, USA), 5 ng/mL human recombinant IL-1β [25,38] (PeproTech, Rocky Hill, SC, USA), 1 µg/mL TLR-3 agonist Poly I/C [32] (Invivogen, San Diego, CA, USA) and 1 µg/mL TLR-2/1 agonist Pam3CSK4 [31,33] (Invivogen, San Diego, CA, USA). Stimulation was performed with 1 mL DMEM per well, supplemented with 1% P/S but without FBS. Unstimulated hPDLSCs served as control. Concerning TLR-2 and TLR-3 agonists, 48 h later, IDO-1 expression was analyzed on gene and protein levels using quantitative chain reaction (qPCR) and immunostaining followed by flow cytometry analysis. Additionally, IDO-1 enzymatic activity was measured by colorimetric enzymatic activity assay.

To investigate the dependency of IL-1β, TNF-α, IFN-γ, Poly I/C and Pam3CSK4 boosted IDO-1 expression on time, the medium of stimulated hPDLSCs was changed to DMEM without any stimuli (supplemented with only 1% P/S) after 48 h. IDO gene expression, the corresponding protein levels and the enzymatic activity was measured at this point (day 0), 24 h (day 1) and 120 h (day 5) later.

### 2.3. 3,4,5-Dimethylthiazol-2-yl-2,5-diphenyl Tetrazolium Bromide Cell Viability Assay

A 3,4.5-dimethylthiazol-2-yl-2,5-diphenyl tetrazolium bromide (MTT) based cellular assay was used to determine the effect of applied stimuli and their removal on the hPDLSCs’ viability. 5 × 10^4^ primary hPDLSCs in 500 µL DMEM, supplemented with 10% FBS and 1% P/S, were seeded per well into 24-well plates. After 24 h of incubation, hPDLSCs were stimulated as described above in 500 µL DMEM, supplemented with only 1% P/S. 48 h later, the medium was changed to DMEM without any stimuli, supplemented with only 1% P/S. Cell viability was measured at this time point and one and five days after removing the stimuli as described in our previous studies [39,40]. In brief, 100 µL MTT solution was added to hPDLSCs followed by two hours incubation at 37 °C. Conditioned medium was removed and insoluble formazan crystals were dissolved in 500 µL dimethyl sulfoxide per well. A microplate reader (Synergy HTX multiplate reader, BioTek, Winooski, VT, USA) was used to measure the absorbance at OD_570 nm_ (optical density) after transferring dissolved crystals into 96-well plates in quadruplets.

### 2.4. qPCR

Lysate preparation of hPDLSCs, mRNA reverse transcription into cDNA and qPCR were performed using TaqMan Gene Expression Cells-to-Ct Kit (Applied Biosystems, Foster City, CA, USA), according to the manufacturer’s instructions. Primus 96 advanced thermocycler (Peq/Lab/VWR, Darmstadt, Hessen, Germany) was used to heat the samples to 37 °C for one hour and to 95 °C for five minutes to perform reverse transcription. qPCR was conducted using Quant Studio 3 (Applied Biosystems, Foster City, CA, USA), heating the samples to 95° Celsius for 10 min followed by 50 cycles of 15 s at 95° Celsius and one minute at 60° Celsius. TaqMan Gene Expression Assays (all from Applied Biosystems, Foster City, CA, USA) were used to quantify IDO-1 and GAPDH gene expression levels: IDO-1, Hs00984148_m1; GAPDH, Hs99999905_m1. GAPDH served as an endogenous reference. The amplification of target genes was performed in paired reactions, followed by the determination of Ct values. The n-fold expression of each target gene compared to the unstimulated control was calculated using the 2^−∆∆Ct^ method.

### 2.5. Intracellular IDO-1 Immunostaining

In total, 250,000 hPDLSCs were harvested and washed with 1 mL 3% bovine serum album (BSA) solution (in 1 × phosphate buffer saline plus 0.09% sodium azid). Fixation and permeabilization of hPDLSCs were performed using the Intracellular Fixation and Permeabilization Buffer Set (eBioscience, Waltham, MA, USA), according to the manufacturer’s guidelines. hPDLSCs were resuspended in 50 µL 1 × Permeabilization buffer and mixed 1:10 with PE-conjugated mouse anti-human IDO-1 antibody (clone eyedio, eBioscience, Waltham, MA, USA) or with PE-conjugated mouse IgG1 immunoglobulin isotype control (eBioscience, Waltham, MA, USA). After 30 min of incubation at room temperature, hPDLSCs were washed with 3% BSA solution two times. IDO-1 intracellular expression was verified by flow cytometry analysis using AttuneTM NxT Flow Cytometry (Thermo Fisher Scientific, Waltham, MA, USA). An argon laser was used to excite the fluorochrome at 488 nm. In total, 10,000 hPDLSCs were acquired per sample to determine the percentage of IDO-1 positive hPDLSCs. AttuneTM NxT Cytometer software (Thermo Fisher Scientific, Waltham, MA, USA) was used for analysis.

### 2.6. IDO-1 Activity Assay

Extra- and intra-cellular IDO-1 activities were determined by measuring L-kynurenine concentrations in CM and cell lysates, respectively. After harvesting CM, hPDLSCs were washed with 1 × PBS followed by incubation for 3 h under standard culture conditions in 500 µL 1 × PBS supplemented with 800 µM L-tryptophan (Sigma-Aldrich, St. Louis, MO, USA). After harvesting hPDLSCs, proteins were precipitated by mixing cell lysates and CM 1:3 (*v*/*v*) with 30% trichloroacetic acid (Sigma-Aldrich, St. Louis, MO, USA), followed by incubation at 65° Celsius for 30 min. After centrifugation, 125 µL supernatants were mixed 1:1 (*v*/*v*) with Ehrlich’s reagent (0.8% P-dimethylbenzaldehyde in acetic acid, Sigma-Aldrich, St. Louis, MO, USA), followed by the photometric measurement of the optical density at 492 nm (OD492). All samples were measured in duplicates. L-kynurenine concentrations were determined by plotting measured OD492 values against a standard curve with known L-kynurenine levels ranging from 1000 µM to 7.8 µM. Calculated L-kynurenine concentrations were normalized to total protein amounts per sample in mg and to the appropriate incubation times in minutes. 

Pierce BCA Protein Assay Kit (Thermo Fisher Scientific, Waltham, MA, USA) was used to determine total protein amounts per sample, following the manufacturer’s guidelines. Measured OD_562 nm_ values were plotted against known BSA levels, which ranged from 2000 µg/mL to 31.25 µg/mL.

### 2.7. Statistical Analysis

All results are presented as mean ± standard error of the mean (s.e.m.). They were obtained from four to eight independent experiments using hPDLSCs isolated from four to eight different individuals. After verifying normal distribution by the Kolmogorov-Smirnov test, one-way analysis of variance (ANOVA) followed by post-hoc test was used to determine statistically significant differences between groups. All statistical analysis was performed using SPSS 24.0 (IBM, Armonk, NY, USA). When showing a *p*-value ≤ 0.05, differences between various groups were considered to be statistically significant.

## 3. Results

**Figure 1:**
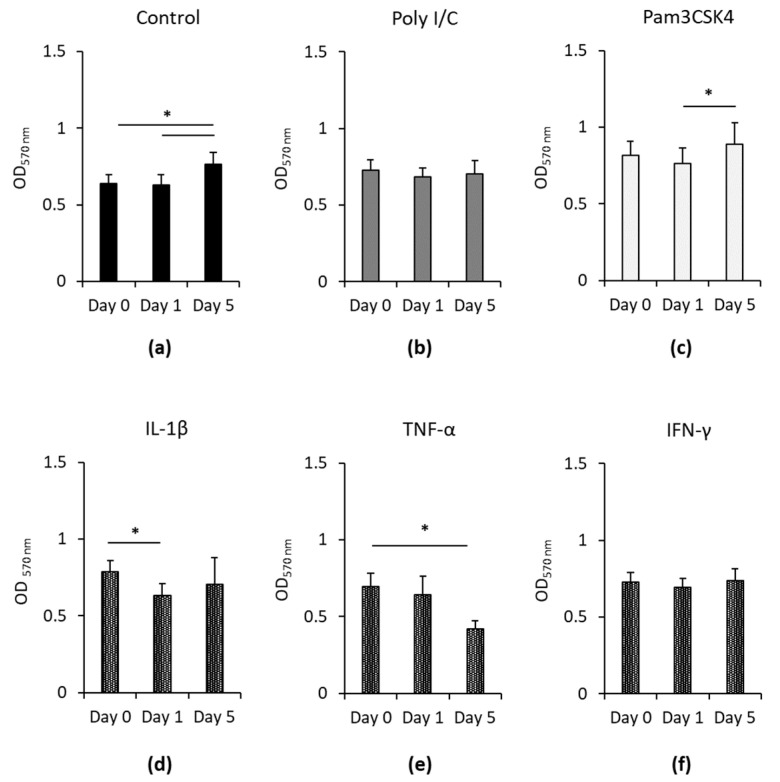
Continuing effect of Poly I/C, Pam3CSK4, IL-1β, TNF-α and IFN-γ on the hPDLSCs’ viability. Primary hPDLSCs were stimulated with 1 µg/mL Poly I/C (**b**), 1 µg/mL Pam3CSK4 (**c**), 5 ng/mL IL-1β (**d**), 10 ng/mL TNF-α (**e**) or 100 ng/mL IFN-γ (**f**) Untreated hPDLSCs served as control (**a**). After 48 h of incubation, the medium was changed to Dulbecco’s modified Eagle’s medium (DMEM) without any inflammatory stimuli. Cell viability was determined by MTT-based photometric assay, immediately and one and five days after removing inflammatory stimuli. The *y*-axis shows the measured absorbance at 570 nm in a linear scale. All data are presented as mean values (±S.E.M.) obtained from six independent experiments using hPDLSCs isolated from six different individuals. * *p*-value ≤ 0.05, significantly different between groups as indicated.

Figure 1 demonstrates the effect of Poly I/C, Pam3CSK4, IL-1β, TNF-α and IFN-γ on the viability of hPDLSCs, one and five days after removing the inflammatory stimuli. Untreated (Figure 1a) or Pam3CSK4 (Figure 1c) stimulated hPDLSCs showed no changes in cell viability one day after removing the stimuli, whereas a significant increase was observed five days after stimuli removal. IL-1β treatment of hPDLSCs (Figure 1d) caused a significant decrease in cell viability one day after the removal of IL-1β. Five days without IL-1β led to an increase in cell viability, however, without any significance. TNF-α treated hPDLSCs (Figure 1e) showed a gradual decrease in cell viability within the observed time frame, causing a significant decline from day zero to five days after removing TNF-α. In Poly I/C (Figure 1b) and IFN-γ (Figure 1f) treated hPDLSCs no changes in cell viability were detected within the observation period.

**Figure 2:**
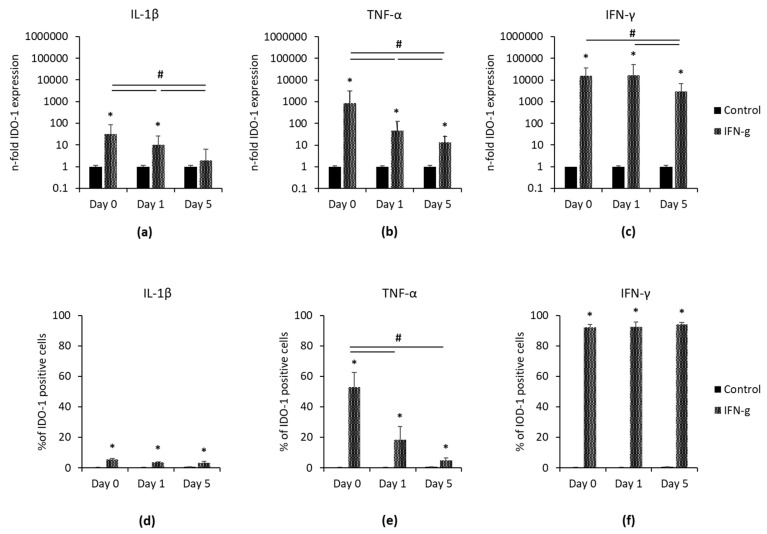
Continuing effect of IL-1β, TNF-α or IFN-γ on IDO-1 expression in hPDLSCs. Primary hPDLSCs were stimulated with 5 ng/mL IL-1β (**a**,**d**), 10 ng/mL TNF-α (**b**,**e**) or 100 ng/mL IFN-γ (**c**,**f**). After 48 h of incubation, the medium was changed to DMEM without any inflammatory stimuli. Gene expression levels (**a**–**c**) and the percentage of IDO-1 positive hPDLSCs (**d**–**f**) were determined by qPCR and intracellular immunostaining followed by flow cytometry analysis, immediately and one and five days after removing inflammatory stimuli. (**a**–**c**) The y-axis shows the n-fold IDO-1 gene expression compared to the appropriate unstimulated control (n-fold IDO-1 expression = 1) in a logarithmic scale, as calculated by the 2^−∆∆Ct^ method. (**d**–**f**) The y-axis shows the percentage of IDO-1 positive hPDLSCs in a linear scale. All data are presented as mean values (±S.E.M.) obtained from five (**a**–**c**) and six (**d**–**f**) independent experiments using hPDLSCs isolated from five or six different individuals, respectively. * *p*-value ≤ 0.05, significantly increased compared to unstimulated control; # *p*-value ≤ 0.05, significantly different between groups as indicated.

Figure 2 demonstrates IDO-1 gene expression and protein levels one and five days after removing IL-1β, TNF-α and IFN-γ. At all investigated time points, TNF-α- and IFN-γ- stimulated hPDLSCs exhibited significantly higher IDO-1 gene expression (Figure 2b,c) compared to the appropriate controls. IL-1β-triggered IDO-1 gene expression (Figure 2a) was also higher compared to the control. However, significant differences were only found at day zero and one day after removing IL-1β. In IL-1β and TNF-α stimulated cells, a gradual, significant decrease of IDO-1 gene expression levels was observed after the removal of corresponding stimuli. In IFN-γ treated hPDLSCs, IDO-1 gene expression level significantly decreased only after five days without IFN-γ. Additionally, all three cytokines significantly increased the percentage of IDO-1 positive hPDLSCs (Figure 2d,f and Supplementary Figure S1) at all time points. In TNF-α stimulated hPDLSCs, a decrease of the percentage of IDO-1 positive cells was observed in a time-dependent manner, showing significant reductions from day 0 to one and five days after the stopping stimulation. IL-1β and IFN-γ triggered hPDLSCs showed no changes in IDO-1 protein production after removing the inflammatory stimuli.

**Figure 3:**
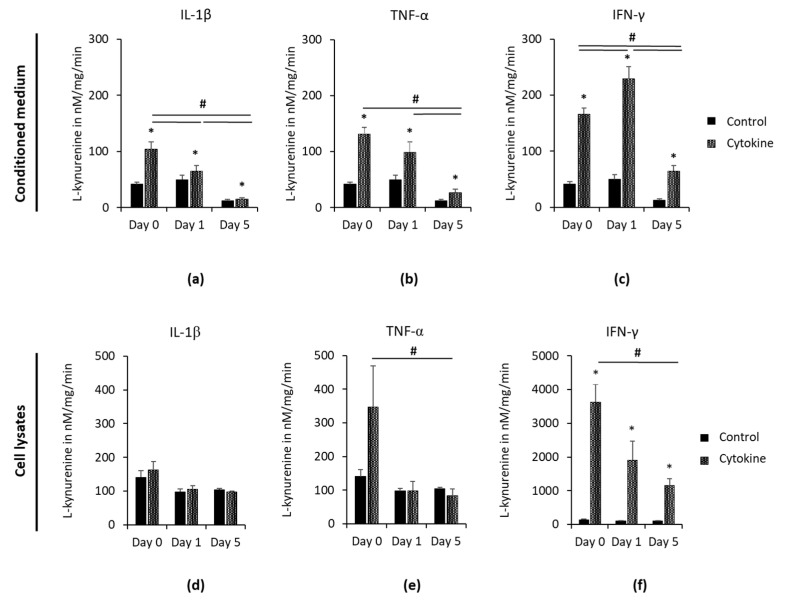
Continuing effect of IL-1β, TNF-α or IFN-γ on IDO-1 enzymatic activity in hPDLSCs. Primary hPDLSCs were stimulated with 5 ng/mL IL-1β (**a**,**d**), 10 ng/mL TNF-α (**b**,**e**) or 100 ng/mL IFN-γ (**c**,**f**). After 48 h of incubation, the medium was changed to DMEM without any inflammatory stimuli. IDO-1 enzymatic activities were calculated via measuring L-kynurenine concentrations in CM (**a**–**c**) and cell lysates (**d**–**f**) immediately and one and five days after removing inflammatory stimuli. Determined L-kynurenine levels were normalized by the total protein amount per sample and the appropriate incubation times. The *y*-axis shows the L-kynurenine concentrations in nM/mg/min in a linear scale. All data are presented as mean (±S.E.M.) obtained from 4 independent experiments using hPDLSCs isolated from 4 different individuals. * *p*-value ≤ 0.05, significantly increased compared to the unstimulated control of the appropriate day; # *p*-value ≤ 0.05, significantly different between groups as indicated.

L-kynurenine concentrations in CM and cell lysates, which were determined at day 0 and one and five days after removing the cytokines, are shown in Figure 3. Independently from time, all three cytokines caused significant higher L-kynurenine concentrations in CM (Figure 3a–c) compared to the appropriate controls. At day 5, IDO-1 enzymatic activity of unstimulated control was lower compared to previous time points, but without any significance. Microscopic observations showed no differences between observed time points, revealing cell layers with 100% density, with fibroblast-like cell morphology and with a neglectable number of cells in suspension. In the presence of IL-1β or TNF-α, a significant decrease of L-kynurenine levels in a time-dependent manner was observed, having the highest and lowest L-kynurenine levels at day 0 and five, respectively. IFN-γ caused a significant enhancement of L-kynurenine concentration from day 0 to one day after removing the stimulus, followed by a significant decrease in L-kynurenine levels at day 5. In cell lysates (Figure 3d,e), IL-1β caused L-kynurenine concentrations comparable to the unstimulated control. TNF-α clearly increased L-kynurenine levels in cell lysates at day 0, but without any significance. This increased L-kynurenine concentrations decreased to levels comparable to the unstimulated control, showing significant differences between day 0 and five. Although IFN-γ caused a decrease of L-kynurenine levels in a time-dependent manner with significantly lower L-kynurenine concentrations at day five compared to day 0, IFN-γ caused significantly increased L-kynurenine levels at all investigated days.

**Figure 4:**
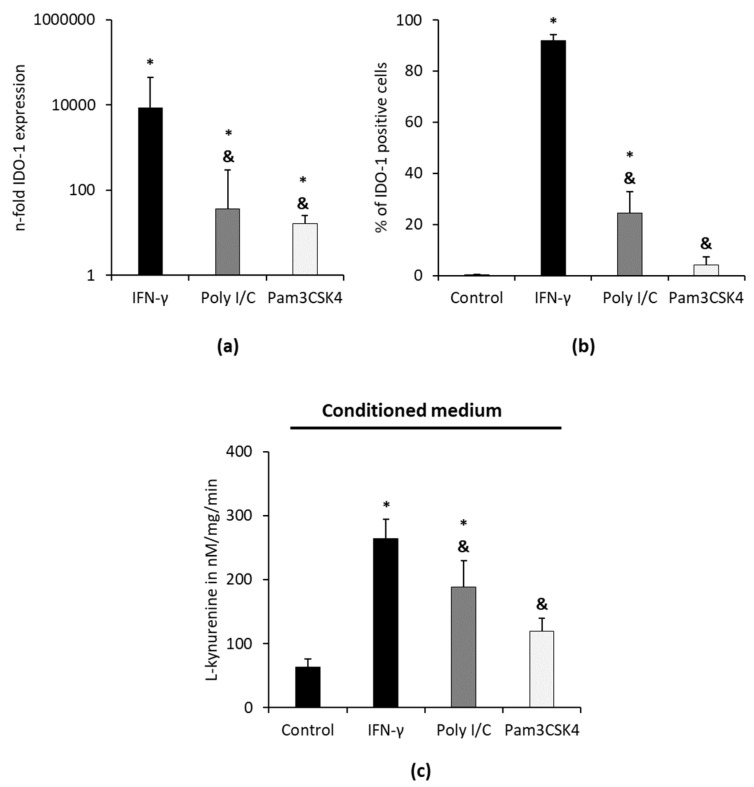
Effect of toll-like receptor (TLR) agonists Poly I/C and Pam3CSK4 on the IDO-1 gene and protein expression and on the enzymatic activity in hPDLSCs. Primary hPDLSCs were stimulated with 1 µg/mL Poly I/C, 1 µg/mL Pam3CSK4 and 100 ng/mL IFN-γ (positive control). After 48 h, IDO-1 gene expression levels (**a**), the percentage of IDO-1 positive cells (**b**) and IDO-1 enzymatic activity (**c**) were determined by qPCR, intracellular immunostaining followed by flow cytometry analysis and by calculating L-kynurenine concentrations in the conditioned media (CM), respectively. (**a**) The y-axis shows the n-fold IDO-1 gene expression compared to the appropriate unstimulated control (n-fold IDO-1 expression = 1) in a logarithmic scale, as calculated by the 2^−∆∆Ct^ method (**b**) The *y*-axis shows the percentage of IDO-1 positive hPDLSCs in a linear scale. (**c**) shows normalized L-kynurenine concentrations in nM/mg/min. All data are presented as mean values (±S.E.M.) obtained from six or eight independent experiments using hPDLSCs isolated from 6 or 8 different individuals, respectively. * *p*-value ≤ 0.05, significantly increased compared to the unstimulated control; & *p*-value ≤ 0.05, significantly decreased compared to IFN-γ stimulated hPDLSCs.

The effect of TLR-3 agonist Poly I/C and TLR-2/1 agonist Pam3CSK4 on IDO-1 gene and protein expression and IDO-1 enzymatic activity in hPDLSC is presented in Figure 4. The exemplary dot plots and gating strategy are presented in Supplementary Figure S1. After 48 h of stimulation, Poly I/C and Pam3CSK4 significantly increased IDO-1 gene expression levels (Figure 4a) and the percentage of IDO-1 positive hPDLSCs (Figure 4b). Poly I/C and Pam3CSK4 caused significantly lower IDO-1 expression compared to the positive control (cytokine IFN-γ) at both gene and protein levels. Poly I/C stimulation increased IDO-1 gene and protein expression levels compared to Pam3CSK4, but without any significance.

Poly I/C but not Pam3CSK4 caused a significant increase in L-kynurenine levels in CM. Both investigated TLR agonists led to significantly lower L-kynurenine concentrations in CM compared to IFN-γ. In cell lysates, used TLR agonists caused L-kynurenine levels below the detection limit (data not shown).

**Figure 5:**
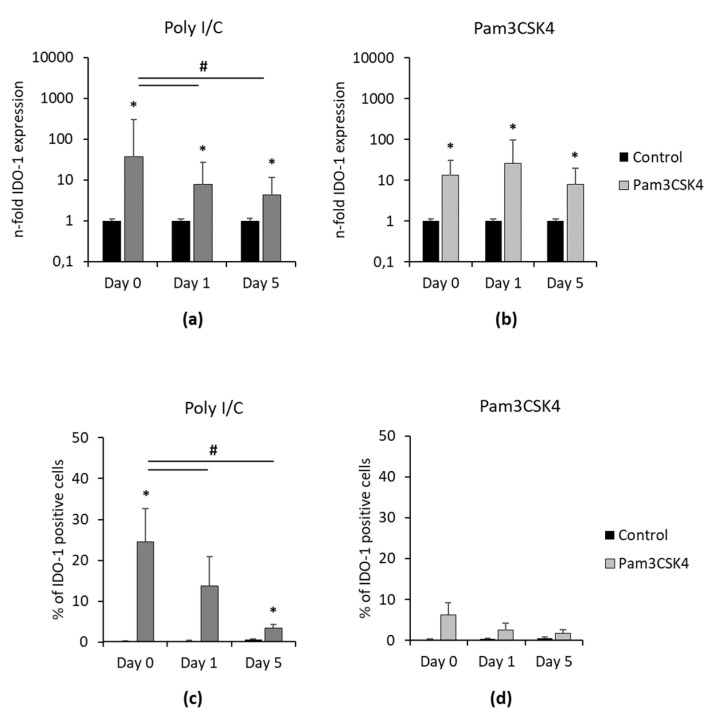
Continuing effect of Poly I/C and Pam3CSK4 on IDO-1 expression in hPDLSCs. Primary hPDLSCs were stimulated with 1 µg/mL Poly I/C (**a**,**c**) or 1 µg/mL Pam3CSK4 (**b**,**d**). After 48 h of incubation, the medium was changed to DMEM without any inflammatory stimuli. Gene expression levels (**a**,**b**) and the percentage of IDO-1 positive hPDLSCs (**c**,**d**) were determined by qPCR and intracellular immunostaining followed by flow cytometry analysis, immediately and one and five days after removing inflammatory stimuli. (**a**,**b**) The *y*-axis shows the n-fold IDO-1 gene expression compared to the appropriate unstimulated control (n-fold IDO-1 expression = 1) in a logarithmic scale, as calculated by the 2^−∆∆Ct^ method. (**c**,**d**) The *y*-axis shows the percentage of IDO-1 positive hPDLSCs in a linear scale. All data are presented as mean values (±S.E.M.) obtained from six independent experiments using hPDLSCs isolated from six different individuals. * *p*-value ≤ 0.05, significantly increased compared to unstimulated control; # *p*-value ≤ 0.05, significantly different between groups as indicated.

Figure 5 shows the IDO-1 gene expression and protein levels one and five days after removing Poly I/C and Pam3CSK4. Independently from time, Poly I/C and Pam3CSK4 significantly increased IDO-1 gene expression (Figure 5a,b) compared to the appropriate controls. In Poly I/C stimulated hPDLSCs, IDO-1 gene expression levels were gradually decreased, showing a significant reduction in IDO-1 gene expression from day 0 to day 1 and 5. In Pam3CSK4 treated hPDLSCs, similar IDO-1 gene expression levels were observed up to five days after Pam3CSK4 removal. The percentage of IDO-1 positive hPDLSCs decreased over time in the presence of Poly I/C, showing significant reductions from day 0 to one and five days without Poly I/C. In Pam3CSK4 treated hPDLSCs, no significant decrease in the percentage of IDO-1 positive cells was observed after the removal of stimuli.

**Figure 6:**
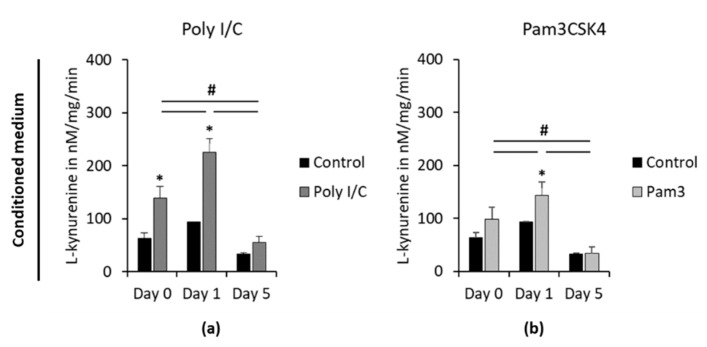
Continuing effect of Poly I/C or Pam3CSK4 on IDO-1 enzymatic activity in hPDLSCs. Primary hPDLSCs were stimulated with 1 µg/mL Poly I/C (**a**) or 1 µg/mL Pam3CSK4 (**b**). After 48 h of incubation, the medium was changed to DMEM without any inflammatory stimuli. IDO-1 enzymatic activities were calculated via measuring L-kynurenine concentrations in CM immediately and one and five days after removing inflammatory stimuli. Determined L-kynurenine levels were normalized by the total protein amount per sample and the appropriate incubation times. The y-axis shows the L-kynurenine concentrations in nM/mg/min in a linear scale. All data are presented as mean (±S.E.M.) obtained from six independent experiments using hPDLSCs isolated from six different individuals. After stimulation of hPDLSCs with all TLR-2 and TLR-3 agonists, L-kynurenine levels in cell lysates were below the detection limit (data not shown). * *p*-value ≤ 0.05, significantly increased compared to unstimulated controls of the appropriate day; # *p*-value ≤ 0.05, significantly different between groups as indicated.

L-kynurenine concentrations in CM, determined at different time points after removing TLR agonists, are shown in Figure 6. At day 0, Poly I/C but not Pam3CSK4 caused significantly higher L-kynurenine concentrations in CM (Figure 6a,b) compared to the appropriate controls. Five days after removing stimuli, IDO-1 enzymatic activity of unstimulated controls was lower compared to previous time points, however without any significance. No differences between observed time points were microscopically detectable. Microscopic observations revealed cell layers with 100% density, fibroblast-like cell morphology and a neglectable number of cells in suspension. One day after removing the stimuli, both TLR agonists exhibited significantly enhanced L-kynurenine concentrations compared to day 0. This was followed by a significant decrease in L-kynurenine levels after 5 days without any stimuli, which was comparable to the appropriate unstimulated control.

## 4. Discussion

hPDLSCs seem to possess a crucial role in regulating the local immuno-inflammatory state and consequently the homeostasis of periodontal tissue. This is executed via their immunomodulatory activities, such as through IDO-1 based paracrine mechanisms, which are affected by several endogenous and exogenous environmental factors [19]. IDO-1 is one of the most investigated immunomediator, which was demonstrated to be one of the key functional immunomediator suppressing various immune cells (reviewed in [19,26]). Additionally, our previous study demonstrated via pharmacological inhibition the functional involvement of IDO-1 in the observed suppression of CD4^+^ T lymphocyte proliferation [25]. These IDO-1 based immunomodulatory mechanisms seem to play crucial roles in both the hPDLSCs’ in vivo regeneration potential and inflammatory processes [18]. Hence, these cells are a promising therapeutic candidate against inflammatory diseases [16] or in regenerative medicine [15].

The used cytokines and TLR-agonists concentrations were mainly chosen due to our experiences from previous studies. 5 ng/mL IL-1β and 100 ng/mL IFN-γ reflect the particular concentration in the gingival crevicular fluid (GCF) of periodontitis-affected patients [41,42]. Additionally, in dose-response experiments from our previous study, these concentrations caused the highest percentage of IDO-1 positive hPDLSCs [25]. 10 ng/mL TNF-α is higher than its concentration (up to 100 pg/mL) in the GCF of periodontitis patients [43]. In the dose-response experiments from our previous study, 10 ng/mL TNF-α caused the highest submaximal percentage of IDO-1 positive hPDLSCs [25]. Additionally, most previous studies used 10 ng/mL TNF-α [37]. 1 µg/mL Poly I/C and Pam3CSK4 were chosen since both TLR-agonists concentrations caused the maximal response in hPDLSCs in dose-response experiments from previous studies [32,33]. It was also shown that 1 µg/mL Poly I/C had no effect on the proliferation of hPDLSCs after stimulation for 48 h [32].

Multiple studies already established that cytokines, such as TNF-α, IL-1β and IFN-γ, highly influence immunomodulatory activities of MSCs isolated from various tissues (reviewed in [19,26]). It is also well demonstrated that cytokines cause high plasticity of immunomodulatory activities in MSCs from various tissues by inducing variable expression of immunomediators, such as IDO-1 [36,37,38,44,45]. Our previous study also showed that TNF-α, IL-1β and IFN-γ affect IDO-1 expression and its enzymatic activity in hPDLSCs in different ways. Additionally, by using pharmacological inhibition of IDO-1, we demonstrated, that the observed plasticity of induced IDO-1 expression has a functional impact on hPDLSCs’ immunomodulatory activities, causing a variable effect on T lymphocyte proliferation [25]. This dynamic responsiveness of hPDLSCs against endogenous inflammatory stimuli may be additionally increased in a temporally dependent manner since it is already known that IDO-1 induction in hPDLSCs varies between 18 and 72 h [31]. Although this study underlines the importance of the time-dependent induction of IDO-1 expression, so far, no study exists which investigates the ongoing effects of various cytokines on IDO-1 production and on its enzymatic activity in hPDLSCs after removing the induction stimuli. Since the ex vivo treatment of hPDLSCs with inflammatory cytokines is a possible new approach to increase the efficacy of MSC-based therapies [27], it is crucial to know how long hPDLSCs can retain the immunomodulatory phenotype after the treatment with inflammatory cytokines. Hence, we explored in this study for the first time the continuing effects of various cytokines on IDO-1 in hPDLSCs one and five days after the withdrawal of any stimuli. Concerning IL-1β and TNF-α, IDO-1 gene and protein levels decreased in a time-dependent manner, showing a significant reduction already one day after removing the stimuli. This time-dependency was also observed by determining its enzymatic activities in CM. In cell lysates, measured L-kynurenine levels were comparable to unstimulated controls, already at day 1. This suggests that hPDLSCs quickly respond to environmental changes and that IL-1β and TNF-α treated hPDLSCs need a continuous trigger to maintain IDO-1 production and its enzymatic activity for a prolonged time. However, it is known that TNF-α causes apoptosis [46], which was indirectly shown in our cell viability assay. Hence, it is possible that the observed reduction in IDO-1 production and activity in TNF-α stimulated hPDLSCs may be caused due to a gradual decrease in the viability of hPDLSCs from day zero to five days after removing the stimulus, rather than from the declining effect of TNF-α on IDO-1.

We further showed for the first time that IFN-γ has a significant long-lasting effect on the IDO-1 gene and protein level and its enzymatic activities in the CM and cell lysates. Our data contradict the conclusion of Grinnemo et al. who demonstrated an immediate reduction of IDO-1 activity in first-trimester human fetal cardiac MSCs two days after removing IFN-γ [47]. These differences may be explained by the use of MSCs from different origins and differently used incubations times in the presence of IFN-γ. Hence, it seems that IFN-γ may be an excellent choice to activate the immunosuppressive phenotype and hold it for a prolonged time, at least in hPDLSCs. These observations could be essential in MSC-based therapies. Since it is known that the therapeutic potential of transplanted MSCs mainly comes from their immunomodulatory activities [18], studies already try to increase their immunomodulatory and therefore their regeneration potential via IFN-γ treatment [27]. Further, our cell viability assay exhibited that, under the investigated cytokines, IFN-γ treated hPDLSCs showed the most robust cell viability within the monitoring period. Hence, these observations suggest that IFN-γ could be a potential candidate to enhance the immunomodulatory activities of hPDLSCs beyond their transplantation.

Translating these data into clinic, it is well known that TNF-α, IL-1β and IFN-γ are abundant in periodontitis-associated lesions [42,48,49,50,51] and are involved in the destructive immune response and in pathogenic alveolar bone remodeling during periodontitis [42,48,49,50,51]. Additionally, our data indicate that these cytokines trigger an immunosuppressive effect, at least partially, via IDO-1 expression in hPDLSCs, counteracting its destructive role during periodontitis. Since cytokine levels vary during periodontal disease progression, the observed temporal reduction of IDO-1 productions and its enzymatic activity may have a physiological significance. It is possible that the time-dependent variable effect of these cytokines on the IDO-1 expression in hPDLSCs may enhance the plasticity of their immunomodulatory activities, which would make these cytokines crucial for fine-tuning the local balanced immuno-inflammatory state in periodontal tissues.

Although the expression of TLRs in hPDLSCs is well investigated, their contribution to immunomodulatory activities of hPDLSCs has to be further clarified [19]. Among TLR-2 and TLR-3 agonists, only Poly I/C significantly increased the percentage of IDO-1 positive hPDLSCs. Furthermore, only Poly I/C induced significantly higher IDO-1 enzymatic activities in CM. These differences may be explained by the MyD88 independent activation of IRF3 transcription factor by TLR-3 agonist Poly I/C, followed by transcription of type I IFN genes [52]. It is already demonstrated that type I IFN, besides IFN-γ, also induces IDO-1 [53]. TLR-2/1 agonist Pam3CSK4, in contrast, activates the MyD88 dependent signaling pathway and consequently NF-κB and AP-1 transcription factors followed by the expression of various pro-inflammatory cytokines [54], which are not known to activate IDO-1 expression.

Only a few previous studies already investigated the influence of TLR-2/1 and TLR-3 agonists on IDO-1 expression in hPDLSCs. In our previous study, we investigated the impact of TLR-2 agonist Pam3CSK4 alone and in combination with IFN-γ on the expression of IDO-1 and several cytokines in hPDLSCs. This study demonstrated a potential interaction between TLR agonists and IFN-γ induced IDO-1 expression in hPDLSCs. Additionally, we demonstrated an increased IDO-1 gene expression in hPDLSCs in the presence of TLR-2 agonist Pam3CSK4, but no effect on IDO-1 protein levels [31], which is in line with the findings of this study. This previous study has investigated the continuing effect of neither TLR agonists nor IFN-γ on IDO-1 expression in hPDLSCs [31]. Further, significant and slightly increased L-kynurenine levels in the CM in the presence of TLR-3 agonist Poly I/C and TLR-2/1 agonist Pam3CSK4, respectively, were also observed by Mahanonda et al., in MSCs isolated from the gingiva [55]. In cell lysates, none of the used TLR agonists triggered IDO-1 enzymatic activity.

Although Pam3CSK4 significantly increases IDO-1 gene expression, this TLR agonist seems to have only a limited potential to trigger IDO-1 protein expression or its enzymatic activity. This decoupling of IDO-1 gene and protein expression was already observed in the literature. Issaranggun et al., reported an increase in IDO-1 gene expression in IL-12 treated hPDLSCs already after 24 h, whereas a 7-day treatment was needed to induce IDO-1 enzymatic activity [56].

Several studies already reported that various TLRs agonists, such as Pam3CSK4 and Poly I/C, significantly induce the expression of various pro-inflammatory cytokines in hPDLSCs [31,32,35]. TLR-3 agonist Poly I/C and TLR-2/1 agonist Pam3CSK4 cause comparable expression of pro-inflammatory cytokines in hPDLSCs, such as IL-6, IL-8 and monocyte chemoattractant protein (MCP)-1 [32]. The present study suggests that in contrast to TLR-3, TLR-2 is a weak activator of immunomodulatory factors, like IDO-1. Therefore, activation of hPDLSCs by TLR-2 agonist might shift the balance toward a more pro-inflammatory state and consequently to tissue destruction.

For the first time, this study investigated the ongoing effect of activated TLR-2 and TLR-3 on IDO-1 production and its enzymatic activity. Interestingly, we have observed a significant increase in IDO-1 enzymatic activity in CM at day 1 after the removal of TLR-2 and TLR-3 agonists compared to day 0. The enzymatic activity further declined to a level comparable to the unstimulated control five days after the withdrawal of TLR-2 and TLR-3 agonists. These data indicate that TLR-2 and TLR-3 agonists also contribute to a high degree of plasticity of the immunomodulatory activities in hPDLSCs. It seems that hPDLSCs react rapidly to the appearance of virulence factors by increasing the expression of immunomodulatory factors and can retain and even enhance this expression shortly after the removal of the antigens. This also points to a high degree of plasticity of hPDLSCs which may be essential to recuperate local periodontal tissue homeostasis during periodontitis.

Several databases, such as UniProt or the Atlas of Genetics and Cytogenetics in Oncology and Haematology, indicate that IDO-1 is an intracellular protein with no secreted or extracellular form, localized in the cytosol. However, there are several hints that IDO-1 can be secreted and detected in the extracellular space, such as from adipose-derived mesenchymal stromal cells [57]. Our results support this fact due to the following observations. Our results indicate clear differences in L-kynurenine levels measured in CM and cell lysates. Moreover, no L-kynurenine was detectable in cell lysates, with the exception of IFN-γ, whereas increased extracellular IDO-1 enzymatic activities were detectable in the presence of all cytokines and in the presence of Poly I/C and Pam3CSK4. This indicates that, under the used in vitro conditions, IDO-1 protein may be secreted to the extracellular space. Future studies should evaluate the physiological role of the released and intracellular IDO-1 in the immunomodulatory function of MSCs generally, and in hPDLSCs particularly.

The study design is limited by the use of a heterogenous hPDLSCs’ population, including various cell types instead of pure stromal cell culture. Further, these cells were isolated only from human PDL. Extracting cells from other oral tissues, such as the gingiva or pulp, could enhance the impact of this study. Additionally, this study is observational. No functional experiments, such as hPDLSCs/immune cells co-culture, were performed to verify the functional importance of the observed effects on IDO-1 expression and its enzymatic activity over time. Finally, this study is limited by its in vitro character. It is hard to extrapolate these results into the in vivo situation, since in the clinical setting, cytokine-treated hPDLSCs are transplanted into patients, which could further impact the continuing effect of the ex vivo stimulation.

## 5. Conclusions

In conclusion, this in vitro study demonstrates that IDO-1 production and its enzymatic activity in hPDLSCs is differently influenced by distinct cytokines and TLR-2 and TLR-3 agonists. Furthermore, a different dynamic of IDO-1 activity was observed after the removal of different stimuli. This observation suggests a high degree of plasticity of the immunomodulatory activities in hPDLSCs, determined quantitatively and temporally by different cytokines and TLR-2 and TLR-3 agonists. This study further demonstrates a quick response of hPDLSCs to local changes in the cytokine and virulence factor milieu. All these together may be important for sustaining or recuperating the local immuno-inflammatory status of healthy or periodontitis-affected periodontal tissues, respectively.

## Supplementary Materials

The following are available online at https://www.mdpi.com/2073-4409/9/12/2696/s1, Figure S1: Representative dot plots show the percentage of IDO-1 positive hPDLSCs compared to the unstimulated control.
