# Supplementary material for: Continuing Effect of Cytokines and Toll-Like Receptor Agonists on Indoleamine-2,3-Dioxygenase-1 in Human Periodontal Ligament Stem/Stromal Cells
Source: Cells. 2020 Dec 16;9(12):2696. doi: 10.3390/cells9122696 (PMC7765527; doi:10.3390/cells9122696)
Supplement: Supplementary file 1 [file cells-09-02696-s001.pdf]

cells

Supplementary Information

Article

Persisting effect of cytokines and toll-like receptor agonists on indoleamine-2,3-dioxygenase-1 in human periodontal ligament stem/stromal cells

Christian Behm[1,2], Alice Blufstein[1], Johannes Gahn[1], Barbara Kubin[1], Andreas Moritz[1], Xiaohui Rausch-Fan[1] and Oleh Andrukhov [1,*]

[1] Division of Conservative Dentistry and Periodontology, University Clinic of Dentistry, Medical University of Vienna, Sensengasse 2a, 1090 Vienna, Austria; christian.behm@meduniwien.ac.at (CB); alice.blufstein@meduniwien.ac.at (AB); Johannes.gahn@gmail.com (JG); andreas.moritz@meduniwien.ac.at (AM); xiaohui.rausch-fan@meduniwien.ac.at (XRF)

[2] Division of Orthodontics, University Clinic of Dentistry, Medical University of Vienna, Sensengasse 2a, 1090 Vienna, Austria; christian.behm@meduniwien.ac.at (CB)

* Correspondence: oleh.andrukhov@meduniwien.ac.at; Tel.: +43(0)1-40070-2620

Supplementary Figure 1

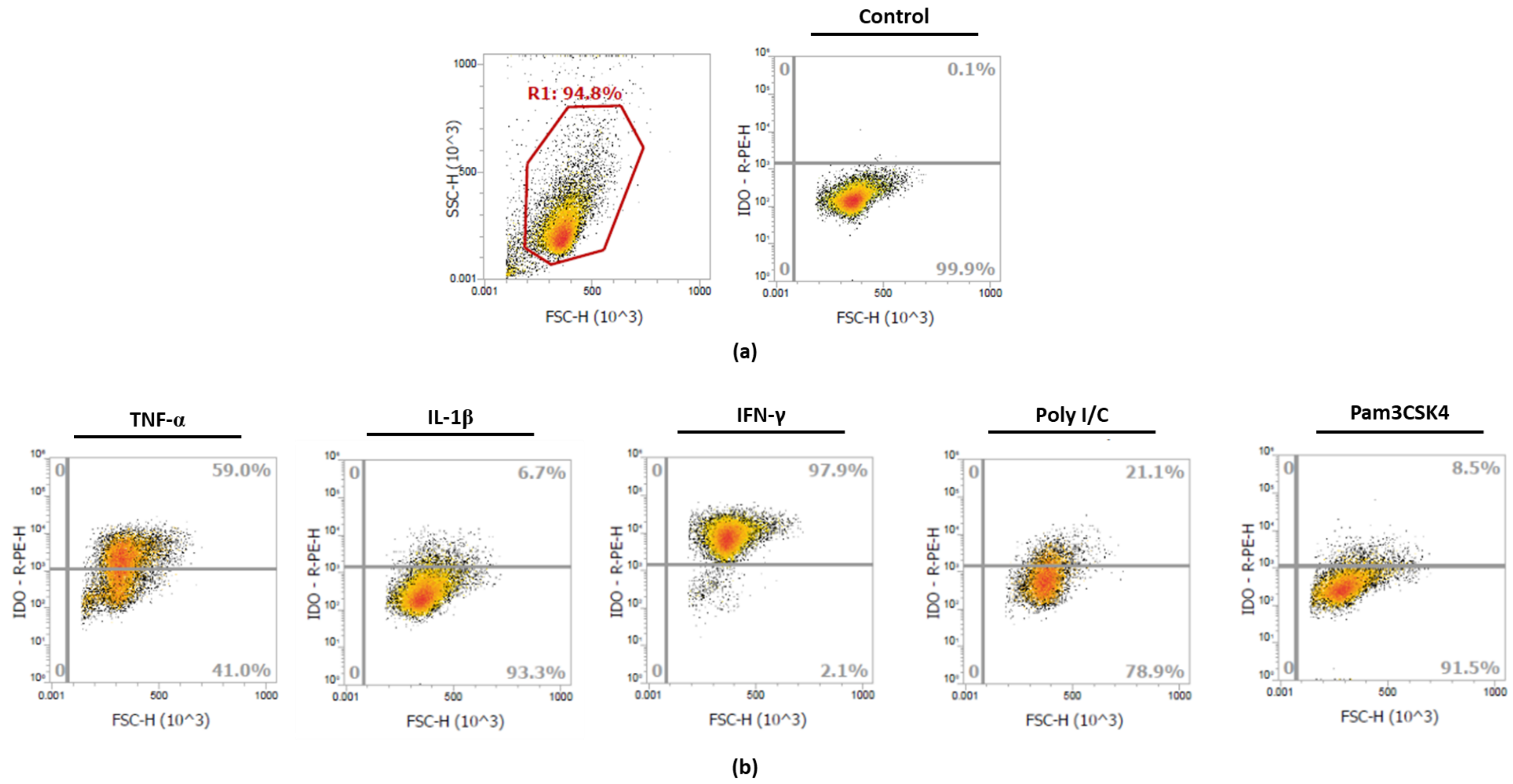

Supplementary Figure 1. Representative dot plots (b) show the percentage of IDO-1 positive hPDLSCs compared to the unstimulated control (a). Primary hPDLSCs were stimulated with 10 ng/ml TNF-α, 5 ng/ml IL-1β, 100 ng/ml IFN-γ, 1 µg/ml Poly I/C or 1 µg/ml Pam3CSK4. After 48 hours incubation, IDO-1 protein levels were determined by intracellular immunostaining followed by flow cytometry analysis.
